# Right-sided Aortic Arch with Aberrant Left Subclavian Artery from Kommerell’s Diverticulum

**Published:** 2011-09-25

**Authors:** M. Y. Mubarak, A. T. Kamarul, M. D. Noordini

**Affiliations:** 1 Radiologist, Department of Diagnostic Imaging, Tengku Ampuan Afzan Hospital, Kuantan, Pahang Darul Makmur, Malaysia; 2 Radiographer, Department of Diagnostic Imaging, Tengku Ampuan Afzan Hospital, Kuantan, Pahang Darul Makmur, Malaysia; 3 Anaesthesiologist, Department of Anaesthesia and Intensive Care, Tengku Ampuan Afzan Hospital, Kuantan, Pahang Darul Makmur, Malaysia

**Keywords:** Right-Sided Aortic Arch, Aberrant Left Subclavian Artery, Kommerell’s Diverticulum

## Abstract

A previously healthy 52-year-old man had a chest radiograph for medical check-up and found to have a right-sided aortic arch. Computed tomography of the thorax revealed a right-sided aortic arch with aberrant left subclavian artery originated from Kommerell’s diverticulum. Barium swallow examination showed compression of the posterior wall of the esophagus. He was asymptomatic and no surgical intervention was performed.

## Introduction

Right-sided aortic arch is a rare anatomical variant present in about 0.1% of the adult population.[1][2] Half of the cases are associated with an aberrant left subclavian artery (0.05%-0.1%). Right-sided aortic arch with aberrant left subclavian artery is less common than left-sided aortic arch with aberrant right subclavian artery (0.5- 2.0%).[3][4] A right-sided aortic arch is an anatomic variant resulting from persistence of the right fourth aortic arch and involution of the left. It can be associated with an aberrant left subclavian artery arises from Kommerell’s diverticulum. It is usually asymptomatic and diagnosed incidentally during adult age.

## Case Presentation

**Fig. 1:**
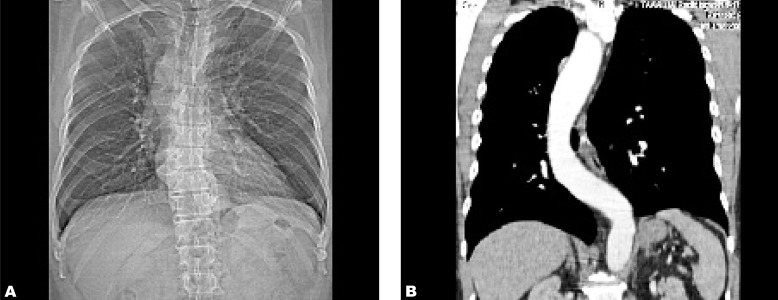
A 52-year-old man with right-sided aortic arch and aberrant left subclavian artery. A. Chest radiograph shows widening of the right mediastinum with right-sided aortic arch. B. Coronal multiplanar reformatted CT image of the right-sided thoracic aorta.

A previously healthy fifty-two year old man had a chest radiograph for his reemployment. The radiograph showed right-sided aortic knob with widening of the right mediastinum. The thoracic aorta was tortuous on the right side of the spine. The normal thoracic aorta contour on the left side was not visualized (Fig. 1). From the chest radiograph, the diagnosis of right-sided aortic arch was made. He had no difficulty in breathing and swallowing.

**Fig. 2:**
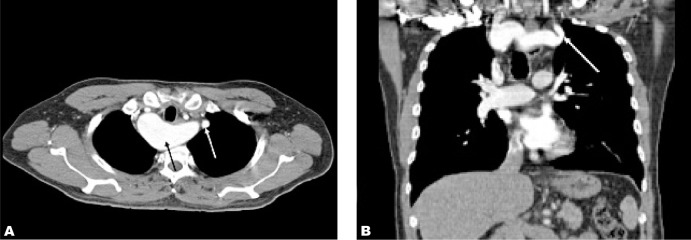
A. Axial CT image of the right-sided aortic arch in the same patient. Kommerell’s diverticulum (black arrow) and origin of aberrant left subclavian artery (white arrow) is seen in this image. B. Coronal multiplanar reformatted CT image of the origin of aberrant left subclavian artery (white arrow).

**Fig. 3:**
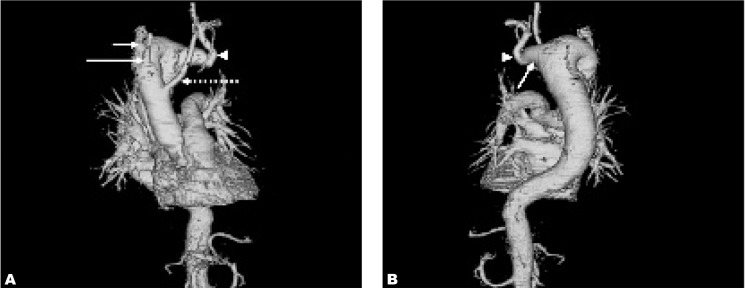
A. Anterior coronal volume rendering image in the same patient shows the arrangement of the arteries from the right-sided aortic arch; left common carotid artery (dashed arrow), right common carotid artery (long arrow), right subclavian artery (short arrow), aberrant left subclavian artery (arrowhead). B. Posterior coronal volume rendering image reveals the Kommerell’s diverticulum (arrow) with aberrant left subclavian artery (arrowhead).

**Fig. 4:**
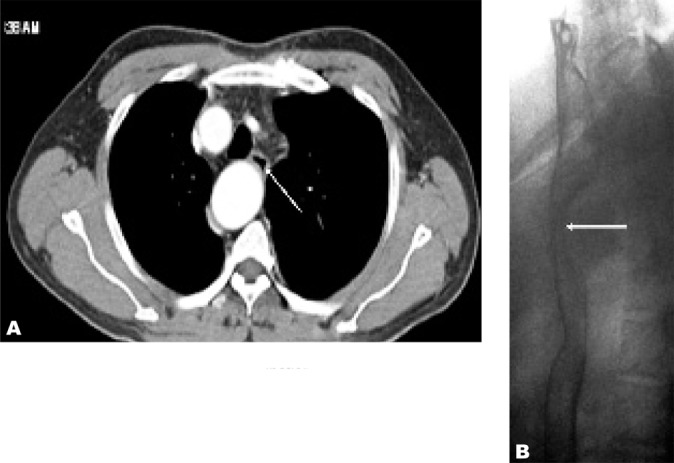
A. Axial CT image shows esophageal compression (arrow) B. Lateral view of barium swallow examination reveals smooth posterior esophageal compression by the aortic arch (arrow).

He had contrast-enhanced computed tomography (CECT) of the thorax to evaluate the thoracic aorta. The right-sided aortic arch was confirmed by the thoracic aorta descending on the right side of the spine then turning to the left to enter the aortic hiatus at normal position (Fig. 1). There was an aberrant left subclavian artery arising from an aortic arch diverticulum (Kommerell’s diverticulum) (Fig. 2). The branches of the right-sided aortic arch from proximal to distal were left common carotid, right common carotid, right subclavian and left subclavian arteries (Fig. 3). The Kommerell’s diverticulum was posterior to the trachea and esophagus. There was compression of the esophagus posteriorly and on the right side (Fig. 4). There was no compression of the trachea. The antero-posterior diameter of the diverticulum was 3.3 cm. The lateral view of barium swallow study revealed a smooth extrinsic compression of the posterior wall of the esophagus (Fig. 4) at the level of the aortic arch and right indentation of the esophagus on the frontal view. Since he was asymptomatic, there was no interventional procedure planned for him. Currently, he is being follow-up by the surgical unit.

## Discussion

Right-sided aortic arch was first documented by Fioratti and Aglietti in 1763.[5] This has been classified by Edward in 1948, Felson and Palayew in 1963 and Steward et al. in 1964.[6] The right-sided aortic arch is classified into three types; type 1, the major arteries branching out from the arch are the left innominate artery, followed by the right common carotid and right subclavian arteries (mirror image branches of normal left aortic arch).[2][7] In type 2, the right-sided arch is found with aberrant left subclavian artery as seen in this case; and in type 3, the left subclavian artery is isolated and does not attach to the aorta (the left subclavian artery is connected to the pulmonary artery through the ductus arteriosus). Type 1 and 2 form 98% of the right-sided aortic arch cases and type 3 is very rare.[8]

There are approximately 50 cases of right-sided aortic arch with aberrant left subclavian artery in the literature.[3] The anomaly is related to the persistence of the right fourth aortic arch and regression of embryonic left fourth arch between the left common carotid artery and left subclavian artery.[9][10] In this anomaly, as also noted in our case, the first trunk branching from the arch was the left common carotid artery which was followed by the right common carotid artery, right subclavian artery and left subclavian artery.[7][11] The branching pattern and radiological features were similarly reported by other authors.[3][4][12][13]

In the literature, it has been reported that the right aortic arch with aberrant left subclavian artery must always have a left ductus arteriosus.[8][14] A vascular ring is formed with the presence of the left ductus arteriosus as it connects the left pulmonary artery to the root of the aberrant left subclavian artery. The vascular ring causes tracheal compression and without tracheal compression or deformity there might be no ring present or the ring does not require treatment.[14] It was one of three most common causes of vascular rings [8][11][12] and in most cases the vascular ring was loose and did not cause compression.[15]

The patient with right-sided aortic arch with aberrant subclavian artery is generally asymptomatic and there is no particular association with cardiac anomalies. [13], [15] There might be symptoms related to the presence of the vascular ring. However, more symptoms are due to atherosclerotic changes of the anomalous vessels, dissection, aneurysm with compression of adjacent structures causing dysphagia (dysphagia lusoria) and dyspnea.[4][9][16][17] In this reported case, there were no symptoms related to the vascular variations and therefore no surgery was indicated.

The aberrant left subclavian artery usually originates from a Kommerell’s diverticulum. The diverticulum is defined as a conical dilatation of the proximal portion of an aberrant subclavian artery near its origin from the aorta.[4][11] It is also known as “lusoria diverticulum”, “remnant diverticulum” or “lusoria root”.[16] It is the remnant of the left fourth aortic arch in the aberrant left subclavian artery though it was originally described by Burckhard Friedrich Kommerell in 1936 [18] in a case of aberrant right subclavian artery (remnant of the right fourth aortic arch) associated with the left aortic arch.[4][12] The location of Kommerell’s diverticulum could be behind the esophagus in 80%, between the trachea and esophagus in 15% and behind the trachea in 5%.[16]
